# Isolation of *rfk-2 ^UV^* , a mutation that blocks spore killing by Neurospora *Spore killer-3*

**DOI:** 10.17912/micropub.biology.000604

**Published:** 2022-07-17

**Authors:** Abraham Velazquez, Elise Webber, Devonte O'Neil, Thomas Hammond, Nicholas Rhoades

**Affiliations:** 1 Illinois State University, School of Biological Sciences, Normal, IL 61790 USA

## Abstract

Neurospora *Spore killer-3* ( *Sk-3* ) is a selfish genetic element that kills spores to achieve gene drive. Here, we describe the isolation and mapping of *rfk-2 ^UV^* , a mutation that disrupts spore killing. The *rfk-2 ^UV^* mutation is located 15.6 cM from *mus-52* on Chromosome III. The significance of this discovery with respect to *Sk-3* evolution is discussed.

**Figure 1. The rfk-2 UV mutation disrupts spore killing and gene drive.**
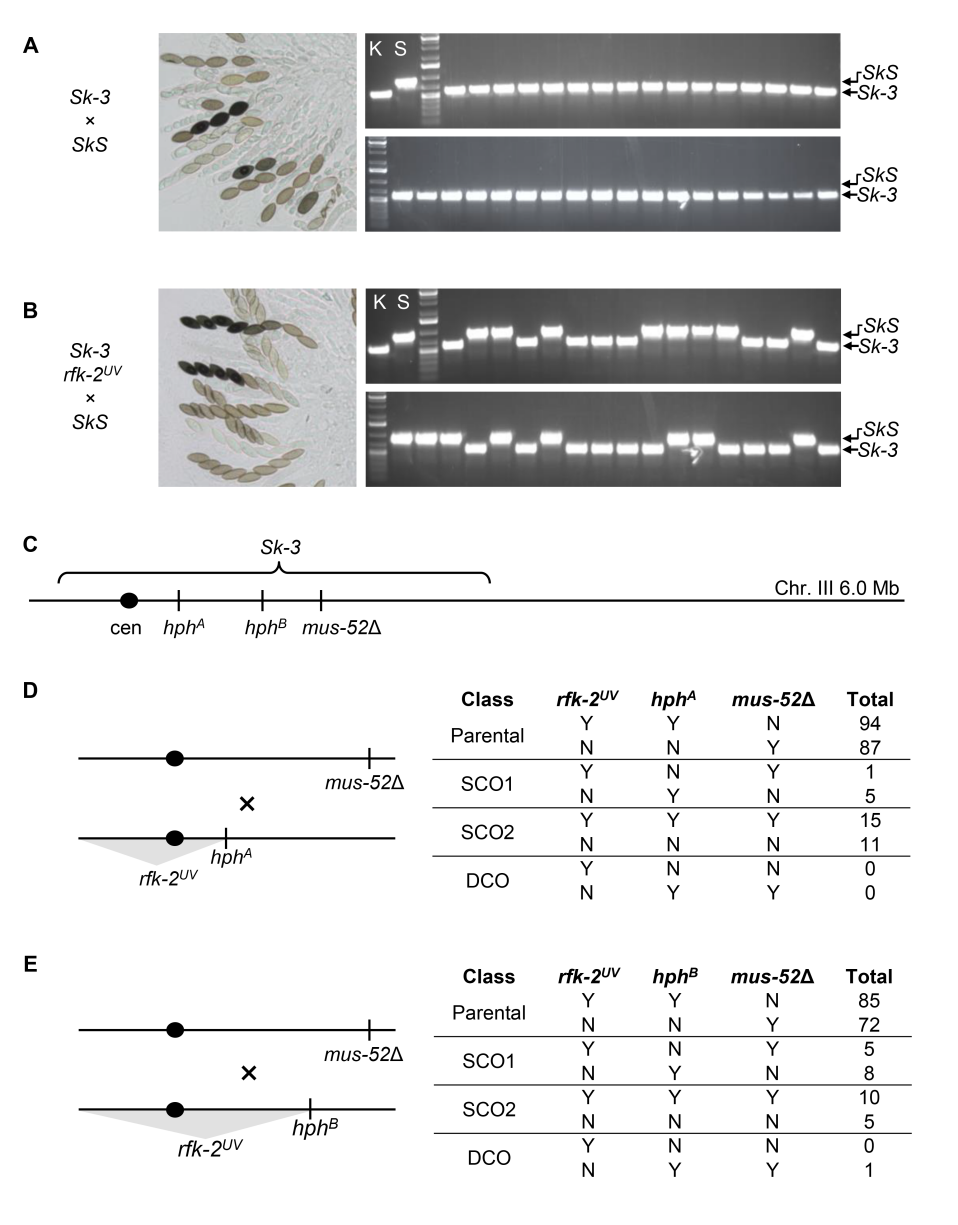
(A) Spore killing and gene drive are present in *SkS* (ISU-3037) × *Sk-3* (ISU-3291) crosses. Left: Asci possess a spore killing phenotype. Asci develop asynchronously in *N. crassa* . Asci with dark pigmented ascospores are more mature than those with light pigmented ascospores. Right: Offspring were randomly collected and examined for *Sk-3* gene drive with a PCR-based gene drive assay. All 34 offspring possess the *Sk-3* genotype, demonstrating that *Sk-3* gene drive occurs in this cross. Lanes: K, *Sk-3* control, ISU-3291; S, *SkS* control, FGSC 10340; two lanes contain a DNA ladder, the remaining 34 lanes correspond to 34 offspring. (B) Spore killing and gene drive are absent from *SkS* (ISU-3036) × *Sk-3 rfk-2 ^UV^* (ISU-4684) crosses. Left: Spore killing is absent from asci. Right: Of the 34 offspring examined, there are 18 with an *Sk-3* genotype and 16 with an *SkS* genotype, demonstrating that *rfk-2 ^UV^* disrupts gene drive (χ ^2^ = 0.12, p-value = 0.73). (C) The *Sk-3* interval is on Chromosome III. The relative positions of the centromere, *hph ^A^* , *hph ^B^* , and *mus-52* Δ are shown in the diagram. (D) Left: A three-point cross was performed with strains *Sk-3 rfk-2 ^UV^ hph ^A^* (ISU-4685/6) and *Sk-3 mus-52* Δ (ISU-4689). Right: A total of 213 offspring were collected and genotyped for *rfk-2 ^UV^ ,* *hph ^A^ ,* and *mus-52* Δ alleles. Recombination analysis indicates that *rfk-2 ^UV^* is located centromere-proximal of *hph ^A^* and *mus-52* Δ. “Y” means that the indicated allele is present in a genotype. Abbreviations: SCO1, genotypes result from a crossover between *rfk-2 ^UV^* and *hph ^A^* ; SCO2, genotypes result from a crossover between *hph ^A^* and *mus-52* Δ; DCO, genotypes result from a double crossover. (E) Left: A three-point cross was performed with strains *Sk-3 rfk-2 ^UV^ hph ^B^* (ISU-4687/8) and *Sk-3 mus-52* Δ (ISU-4689). Right: A total of 186 offspring were collected and genotyped for *rfk-2 ^UV^ ,* *hph ^B^ ,* and *mus-52* Δ alleles. Recombination analysis indicates that *rfk-2 ^UV^* is located centromere-proximal of *hph ^B^* and *mus-52* Δ.

## Description

*Spore killer-3* ( *Sk-3* ) is a selfish genetic element that was discovered over four decades ago in the filamentous fungus *Neurospora intermedia* (Turner and Perkins 1979). *Sk-3* is interesting because it is transmitted to nearly all offspring of an *Sk-3* × *SkS* cross, where *Sk-3* refers to a strain carrying the selfish genetic element, and *SkS* refers to a strain that is sensitive to *Sk-3* -based spore killing. The biased transmission of *Sk-3* is an example of gene drive that occurs through spore killing (Zanders and Johannesson 2021). Specifically, during an *Sk‑3* × *SkS* cross, spore killing eliminates ascospores (offspring) with an *SkS* genotype while sparing those with an *Sk‑3* genotype. As a result, *Sk-3* × *SkS* crosses produce asci with four viable and four inviable ascospores, rather than the eight viable ascospores typical of Neurospora crosses.

*Sk-3* has been mapped to a 30 cM interval of Chromosome III (Turner and Perkins 1979). This interval contains hundreds of genes and it is transmitted to offspring as a single unit (Campbell and Turner 1987). At least two genes within the interval are thought to mediate gene drive. One gene is *rsk,* which is required for resistance to spore killing but not for spore killing itself (Hammond *et al.* 2012). The second gene has yet to be identified, but it is believed to encode *Sk‑3* ’s killer (Hammond *et al.* 2012).

*Sk‑3* is one of two complex selfish genetic elements known to exist in Neurospora fungi. The second is *Sk‑2* (Turner and Perkins 1979). *Sk‑2* shares many similarities with *Sk‑3* . *Sk-2* is transmitted to offspring in a biased manner, resides on a similar interval of Chromosome III, and uses *rsk* for resistance to spore killing but not spore killing itself. Despite these similarities, *Sk‑2* and *Sk-3* are distinct elements. For example, *Sk-2* ’s *rsk* allele ( *rsk ^Sk‑2^* ) provides resistance to spore killing by *Sk-2* but not *Sk-3* , and *Sk-3* ’s *rsk* allele ( *rsk ^Sk‑3^* ) provides resistance to spore killing by *Sk‑3* but not *Sk-2* (Hammond *et al.* 2012).

A recent finding suggests that some of the similarities between *Sk-2* and *Sk-3* , such as their complex genomic rearrangements, may have evolved by convergent evolution (Svedberg *et al.* 2018). Other similarities, such as the role of *rsk* in the drive mechanisms of both *Sk-2* and *Sk-3* , appear to be the result of descent from a common ancestral selfish genetic element. However, a complete understanding of the evolutionary relationship between *Sk-2* and *Sk-3* will likely require additional knowledge, such as the identity of *Sk-3* ’s killer. The *Sk-2* killer is encoded by *rfk-1* and spore killing is absent in *Sk‑2* *rfk-1* Δ × *Sk ^S^* crosses (Rhoades *et al.* 2019). In contrast, deletion of the most likely *rfk-1* ortholog from an *Sk-3* strain had no effect on spore killing, leaving the identity of *Sk-3’s* killer unknown (Svedberg *et al.* 2018).

Here, to help identify *Sk-3* ’s killer, we performed a genetic screen for *required for killing* ( *rfk* ) mutations (see methods). The genetic screen uses *Sk‑3* *rsk* Δ × *SkS* crosses, which abort development before the production of viable ascospores (Hammond *et al.* 2012; Harvey *et al.* 2014). We isolated a few candidate *rfk* mutations with our genetic screen and chose the most promising candidate, *rfk-2 ^UV^* , for additional analysis. As demonstrated in Figure 1 (A and B), *rfk-2 ^UV^* disrupts spore killing and gene drive.

To determine the approximate genomic location of *rfk-2 ^UV^* , we performed two sets of three-point crosses (Figure 1C). Recombination analysis of 213 offspring from the first set of crosses ( *rfk-2 ^UV^* *hph ^A^* × *mus-52* Δ) indicates that *rfk-2 ^UV^* is located 2.8 cM from *hph ^A^* and 15.0 cM from *mus-52* Δ (Figure 1D). For the second set of crosses ( *rfk-2 ^UV^* *hph ^B^* × *mus-52* Δ), recombination analysis of 186 offspring indicates that *rfk-2 ^UV^* is located 7.5 cM from *hph ^B^* and 16.1 cM from *mus-52* Δ** (Figure 1E).

In addition to providing genetic distances from physical positions on *Sk-3* Chromosome III, our recombination data indicate that *rfk-2 ^UV^* is located centromere-proximal of *hph ^A^* , *hph ^B^* , and *mus‑52* Δ (Figure 1, D and E). This is somewhat surprising given that *Sk-2 rfk-1* is located at the junction of *Sk-2* and *SkS* sequences on the right arm of Chromosome III (Rhoades *et al.* 2019). *rfk-1’s* location within *Sk-2* allows it to escape inactivation by a genome defense process called meiotic silencing by unpaired DNA (MSUD) (Aramayo and Selker 2013; Hammond 2017; Rhoades *et al.* 2019), and thus, given the importance of *rfk-1* 's location, we initially predicted that *Sk-3* 's killer gene would be found centromere-distal of *hph ^A^* , *hph ^B^* , and *mus‑52* Δ. Our finding that *rfk-2 ^UV^* is centromere-proximal to all three of these genetic markers indicates that *Sk-2* and *Sk-3* may have evolved different relative positions for their killer genes. In summary, the future cloning and characterization of *rfk-2 ^UV^* should help clarify the organizational patterns of critical gene drive genes within *Sk-2* and *Sk-3* , as well as the evolutionary relationship between these two complex selfish genetic elements.

## Methods

**Strains and alleles used in this study**

*Sk-3* was introgressed into *N. crassa* for genetic analysis shortly after its discovery in *N. intermedia* (Turner and Perkins 1979). Only *N. crassa* strains were used in the present study. The *rid* genotype suppresses a genome defense process called RIP, which mutates duplicated sequences during sexual reproduction (Freitag *et al.* 2002; Aramayo and Selker 2013). *mus-51 ^RIP70^ , mus-51* Δ, and *mus-52* Δ alleles suppress NHEJ, thereby increasing the efficiency of genetic transformation (Ninomiya *et al.* 2004). The *sad-2* Δ allele inhibits MSUD, which suppresses the expression of unpaired genes during meiosis (Aramayo and Selker 2013; Hammond 2017). The *his-3* and *leu-1* genes are required for histidine and leucine biosynthesis, respectively, and *fl* controls macroconidiation (Perkins *et al.* 2000).

**Culture conditions and ascus analysis**

Vegetative propagation was performed on VMM/VMA and crosses were performed on SCA as previously described (Harvey *et al.* 2014; Rhoades *et al* . 2020). For imaging asci, syringe needles were used to dissect asci from perithecia into 50% glycerol at 18 days post fertilization. Asci were imaged by standard light microscopy.

**Screen for *Sk-3* *rfk* mutations**

To isolate *Sk‑3 rfk‑2 ^UV^* , we made one change to a previously developed screen for *Sk-2 rfk* mutations (Harvey *et al.* 2014). Specifically, we irradiated *Sk‑3 rsk* Δ conidia (from strain ISU-4677) instead of *Sk-2 rsk* Δ conidia. We then followed the protocol as previously described by fertilizing *Sk ^S^* protoperithecia with the UV irradiated conidia, incubating the mating cultures for four weeks, collecting shot ascospores from the lids of crossing plates, geminating ascospores on VMA, and transferring individual germlings (offspring) to culture tubes containing VMA for vegetative propagation. Each offspring was genotyped for *Sk-3* and examined for an ability to kill spores in crosses with an *Sk ^S^* mating partner (strains F2-23, F2-26, ISU-3036, and/or ISU-3037). Offspring with an *Sk-3* genotype that displayed defects in spore killing were considered *rfk* mutant candidates. The *rfk-2 ^UV^* mutation was first identified in strain MAV214. The following series of crosses was used to move *rfk-2 ^UV^* from MAV214 into strain ISU-4684: Cross 1) MAV214 × ISU-3036 = ISU-4678; Cross 2) ISU-4678 × ISU-4679 = ISU-4681; Cross 3) ISU-4681 × F2-23 = ISU-4682; Cross 4) ISU-4682 × ISU-3291 = ISU-4683; and, Cross 5) ISU-4683 × ISU-3291 = ISU-4684.

**Genetic modifications**

A standard electroporation-based transformation procedure was used to make genetic modifications to *N. crassa* (Margolin *et al.* 1997; Rhoades *et al.* 2020). All transformation vectors were constructed by double-joint (DJ)-PCR (Yu *et al.* 2004; Hammond *et al.* 2011), using oligonucleotide PCR primers. *Sk-3* genomic DNA was used for amplification of left and right DJ-PCR fragments. The genome sequence of *Sk-3* strain FGSC 3194 (Svedberg *et al.* 2018) was used for primer design and Chromosome III position information. Plasmid pTH1256.1 was used for amplification of *hph* center fragments for DJ-PCR (GenBank: MH550659.1). Plasmid pNR28.12 was used for amplification of *nat* center fragments for DJ-PCR (GenBank: MH553564.1). Both plasmids can be obtained from the Fungal Genetics Stock Center (McCluskey *et al.* 2010). Strain ISU-3291 and transformation vectors v14b, v260, and v337 were used to create *rsk* Δ *::hph* , *leu-1* Δ *::nat* , and *mus-52* Δ *::nat* alleles, respectively. Strain ISU-4684 and transformation vectors v322 and v324 were used to create *hph ^A^* and *hph ^B^* alleles, respectively. Primers for v14b construction: 1001b/1002b (center), 1003b/1004b (left), 1005b/1006b (right), and 1007b/1008b (nested). Primers for v260 construction: 297/298 (center), 1907/1908 (left), 1909/1910 (right), and 1911/1912 (nested). Primers for v322 construction: 585/586 (center), 2204/2158 (left), 2159/2160 (right), and 2161/2162 (nested). Primers for v324 construction: 585/586 (center), 2169/2170 (left), 2171/2172 (right), and 2173/2174 (nested). Primers for v337 construction: 297/298 (center), 2205/2214 (left), 2215/2208 (right), and 2216/2217 (nested).

**PCR-based assay for gene drive**

Genomic DNA was isolated from offspring and control strains with IBI Scientific’s mini genomic DNA kit for plants and fungi. PCR primer set 49/50 amplifies a 596 bp product from *Sk-3* genotypes and an 896 bp product from *SkS* genotypes. PCR products were examined by standard agarose-gel electrophoresis with ethidium bromide staining.

**Three-point crosses**

The position of *rfk-2 ^UV^* was mapped relative to three markers: *hph ^A^* , *hph ^B^* , and *mus-52* Δ. *hph ^A^* was created by inserting the hygromycin resistance cassette ( *hph* ) between genes *ncu05694* and *ncu05695* at approximately 1.0 Mb on Chromosome III of ISU-4684. *hph ^B^* was created by inserting *hph* between genes *ncu07875* and *ncu07876* at approximately 1.6 Mb on Chromosome III of ISU-4684. *mus-52* Δ was created by replacing *mus-52* in strain ISU-3291 with *nat* , a nourseothricin resistance cassette. Offspring were genotyped for *hph ^A^* or *hph ^B^* with hygromycin resistance assays, for *mus-52* Δ with nourseothricin resistance assays, and for *rfk-2 ^UV^* with spore killing assays.

## Reagents

**Table:** 

Strain	Genotype	Source
F2-23	*rid; fl A*	Hammond *et al.* 2012
F2-26	*rid; fl a*	Hammond *et al.* 2012
F3-14	*rid; fl; Sk-3 A*	Hammond *et al.* 2012
FGSC 10340	* rid; mus-51 ^RIP70^ a *	Smith *et al.* 2016
ISU-3036	*rid; fl; sad-2* Δ *::hph A*	Samarajeewa *et al.* 2014
ISU-3037	*rid; fl; sad-2* Δ *::hph a*	Samarajeewa *et al.* 2014
ISU-3291	*rid; Sk-3; mus-51* Δ *::bar A*	P8-42 × F3-14
ISU-4677	*rid; Sk-3 rsk* Δ *::hph; mus-51* Δ *::bar A*	Trans. ISU-3291 with *v14bc*
ISU-4678	*rid; Sk-3 rsk* Δ *::hph rfk-2 ^UV^ ; mus-51Δ::bar; sad-2* D *::hph a*	ISU-4773 *×* ISU-3036
ISU-4679	*rid; Sk-3 leu-1* Δ *::nat-1; mus-51* Δ *::bar A*	Trans. ISU-3291 with v260
ISU-4681	* rid; Sk-3 rfk-2 ^UV^ ; mus-51Δ::bar; sad-2* ^D^ *::hph a*	ISU-4678 × ISU-4679
ISU-4682	* rid; Sk-3 rfk-2 ^UV^ a *	ISU-4681 × F2-23
ISU-4683	* rid; Sk-3 rfk-2 ^UV^ a *	ISU-4682 × ISU-3291
ISU-4684	* rid; Sk-3 rfk-2 ^UV^ ; mus-51* Δ *::bar a*	ISU-4683 × ISU-3291
ISU-4685	* rid; Sk-3 rfk-2 ^UV^ v322* Δ:: *hph; mus-51* Δ *::bar a*	Trans. ISU-4684 with v322
ISU-4686	* rid; Sk-3 rfk-2 ^UV^ v322* Δ:: *hph; mus-51* Δ *::bar a*	Trans. ISU-4684 with v322
ISU-4687	* rid; Sk-3 rfk-2 ^UV^ v324* Δ:: *hph; mus-51* Δ *::bar a*	Trans. ISU-4684 with v324
ISU-4688	* rid; Sk-3 rfk-2 ^UV^ v324* Δ:: *hph; mus-51* Δ *::bar a*	Trans. ISU-4684 with v324
ISU-4689	*rid; Sk-3 mus-52* Δ *::nat; mus-51* Δ *::bar A*	Trans. ISU-3291 with v337
MAV214	*rid; Sk-3 rsk* Δ *::hph rfk-2 ^UV^ ; mus-51Δ::bar a *	ISU-4677-UV × F2-26
P8-42	*rid his-3; mus-51* Δ *::bar a*	Hammond *et al.* 2011
		
PCR Primer Number	Sequence	
49	CCGCTGGTTTGTGGTTCTTGATG	
50	CAGCCACGGATCGCTTATCGTTT	
297	GAGGGAGTGTGGGAAATGGTGTC	
298	GTTGGTTAGGTGGGAACGCTTGT	
585	CCGTCCACGCCCTTAATACGACT	
586	CTTGATTGACAGCGAACGAAACC	
1001b	CTCTGCTCTTCTTTCCCCGCTCCAACTGATATTGAAGGAGCAT	
1002b	AACCTCGATCTCAAATGAAGCCGCAACTGGTTCCCGGTCGGCAT	
1003b	ATAGGGGTGAAAAAGTGGCCTTC	
1004b	ATGCTCCTTCAATATCAGTTGGAGCGGGGAAAGAAGAGCAGAG	
1005b	ATGCCGACCGGGAACCAGTTGCGGCTTCATTTGAGATCGAGGTT	
1006b	CCAGGCACCATCCAAGACAGTT	
1007b	CTGGTCGCTTTTTGCTCTGTTTTCC	
1008b	GTAATTCCAGGTGCCCAAGCTCA	
1907	TGGGTGAATGTCTTGGGAAAGGA	
1908	TGAATGCTAAAAGACACCATTTCCCACACTCCCTCGCTTCGAGGAGCTGGAATTATCAAA	
1909	GCTGGCTGCAATACAAGCGTTCCCACCTAACCAACGGGCGATGCAAACAATGCTCTTT	
1910	CACCTCACATCACACGCTCACCT	
1911	GGACCTCGGGCAAGGATTGTAAG	
1912	CTTTTCCCAAACTGCTCGCTCCT	
2158	AGTCGTATTAAGGGCGTGGACGGCCGACGGATTTAGAACGAGGGC	
2159	GGGTTTCGTTCGCTGTCAATCAAGTCCCCGAAGATAATACCCAAAGAGT	
2160	AGTTTAGAAAACGGCGGCGGAG	
2161	GGCAGAGTGGGTCCTAGCGATA	
2162	AGGGTAAAATGTACGGACGAAGCT	
2169	GGCGACTGTGGAATGGTAAGCG	
2170	AGTCGTATTAAGGGCGTGGACGGCGTAGTGTAGGAAGCTCGGTCA	
2171	GGGTTTCGTTCGCTGTCAATCAAGAGAAATGAGGCTGATAGGTAGACGT	
2172	TTGACCCGACGTTCAAGATGCA	
2173	TGCATTCGACTCACTTGGCATGG	
2174	ACATCTTGCTGCTTCATTTCCCCT	
2204	TTTCAATTTGGGAGCCGGGACTT	
2205	GGGTATGTCAGGGCAAGAACGAC	
2208	GCGTAATTGAGAGGCTCCCAACA	
2214	TGAATGCTAAAAGACACCATTTCCCACACTCCCTCTCATTCGCGGTGGATTTCTAGGC	
2215	GCTGGCTGCAATACAAGCGTTCCCACCTAACCAACTTCAAGAATGTCGAAGGCTGCCA	
2216	GAGAATTGCGGGCGGGGAAGGAC	
2217	GCCCCACTGTAGAGTTCACAAAGGACG	
